# Cosmetic Principles and Contemporary Techniques: Achieving Aesthetic Outcomes in DIEP Flap Breast Reconstruction

**DOI:** 10.3390/jcm15124838

**Published:** 2026-06-22

**Authors:** Christodoulos Kaoutzanis, Bilal F. Hamzeh, Markos Mardourian, David W. Mathes, Julian Winocour

**Affiliations:** Division of Plastic and Reconstructive Surgery, Department of Surgery, University of Colorado Anschutz Medical Campus, Aurora, CO 80045, USA; bilal.hamzeh@cuanschutz.edu (B.F.H.); markos.mardourian@cuanschutz.edu (M.M.); david.mathes@cuanschutz.edu (D.W.M.); julian.winocour@gmail.com (J.W.)

**Keywords:** breast reconstruction, DIEP flap, autologous reconstruction, aesthetic outcomes, nipple-sparing mastectomy, fat grafting, neurotization, abdominal wall morbidity

## Abstract

The deep inferior epigastric perforator (DIEP) flap holds its place as the gold standard approach for autologous tissue breast reconstruction given the strong durability, favorable donor site morbidity, and high patient satisfaction overall. With the reliability and safety of microsurgical reconstruction of the breasts being well-established over these last decades, the goals of DIEP flap reconstruction have expanded beyond flap survival toward optimization of aesthetic, patient-reported, and quality-of-life outcomes. Achieving ideal cosmesis requires thoughtful decision-making across the reconstructive continuum, including of radiation timing, mastectomy incision design, nipple–areolar complex management, reconstructive sequencing, flap shaping and inset, abdominal closure, neurotization, and the potential role of any revision or adjunctive procedures. Modern techniques including delayed-immediate reconstruction, nipple delay, free nipple grafting, fat grafting, and abdominal wall reinforcement have expanded the availability of personalized care in breast reconstruction. This narrative review integrates a targeted literature search with consensus-driven expert opinion informed by our senior authors’ extensive cumulative experience performing DIEP flap breast reconstruction. It discusses principles, technical strategies, and evolving evidence for optimizing aesthetic outcomes in DIEP flap breast reconstruction while preserving safety and minimizing morbidity.

## 1. Introduction

Female breast cancer is among the most commonly diagnosed malignancies worldwide, leading to 2.3 million new cases and 670,000 deaths worldwide in 2020, with a global burden of over 3 million new cases and 1 million deaths projected to occur annually by 2040 [1,2,3]. Today, the prognosis of female breast cancer is favorable. Overall global breast cancer survival has consistently improved, with most regions showing 5-year survival rates above 80% and the highest age-standardized 5-year survival reported in the United States at 90.2% [4]. Of this growing proportion of women diagnosed with breast cancer, data show that as many as 41.5% of certain cohorts undergo mastectomy and have a 10-year overall survival of 72.3% [5]. Among those undergoing mastectomy, as many as 63.4% undergo breast reconstruction, with an upward trend in the utilization of autologous-based breast reconstruction [6]. Autologous breast reconstruction has been shown to yield higher patient satisfaction than implant-based reconstruction and is the preferred option for patients undergoing adjuvant radiation therapy [7,8]. The deep inferior epigastric perforator (DIEP) free flap is considered the gold standard autologous reconstruction option following mastectomy, with favorable postoperative outcomes and rates of flap loss as low as 2.0% [9,10]. Autologous breast reconstruction has been shown to yield excellent patient-reported outcome measures including patient satisfaction and sexual, psychosocial, and physical well-being [11,12].

The treatment of breast cancer is physically and emotionally taxing and is associated with significant psychosocial morbidity. For many patients, breast reconstruction represents one of the few restorative and empowering experiences in an otherwise arduous and burdensome path of cancer treatment, highlighting the distinct privilege and responsibility of the plastic surgeon. Innovation and artistic prowess in plastic and reconstructive surgery have led to significant advancements in aesthetic outcomes following DIEP flap reconstruction. Whereas historically, the primary outcome pursued has been flap survival, global advancements in breast reconstruction over the past two decades have shifted focus toward maximizing comfort, satisfaction, and aesthetic results for patients. In the past, most of the literature was mainly focused on the anatomical and technical aspects of autologous breast reconstruction with DIEP flaps, with limited information about the methods that a surgeon could utilize to maximize aesthetic outcomes. The lack of widespread literature on the analytical approach to achieving optimal aesthetic outcomes made it more challenging for surgeons, especially in the early stages of their careers, to produce reliable and reproducible aesthetic results. The recent literature has explored the role of perforator selection, flap positioning, donor site approaches, autologous fat grafting, and various other techniques to maximize aesthetic outcomes [13,14,15,16,17,18,19]. Blondeel and his colleagues were the first to underscore this shift through their landmark articles discussing their three-step principle for creating aesthetically pleasing breasts in reconstructive breast surgery. The “Easy Three Step Principle” for breast shaping, particularly relevant in breast reconstruction with DIEP flaps, focused on three distinct anatomical concepts to optimize aesthetic outcomes. These included determining the correct breast footprint on the chest wall, designing the appropriate parenchyma volume and projection (conus), and tailoring the skin envelope over the reconstructed conus. This systematic approach aimed to assist surgeons in sculpting DIEP flaps by ensuring the flap’s volume is distributed evenly over the footprint and by maintaining appropriate tension within the skin envelope to produce an aesthetically pleasing reconstructed breast [20,21]. These authors laid the foundation for the subsequent shift in focus observed in the literature regarding DIEP flaps. The focus has since shifted to optimizing the aesthetic outcomes of DIEP flaps as one of the primary goals of reconstruction. Some surgeons have since shared their experiences in the literature by providing important tips throughout each phase of the reconstruction process, expanding our armamentarium with respect to the aesthetic outcomes of DIEP flaps.

This manuscript aims to review the general principles of cosmesis in DIEP flap reconstruction and provide best-practice guidelines by combining a targeted synthesis of the current literature with consensus-driven recommendations provided by our senior authors’ extensive experience performing DIEP flap breast reconstruction at a high-volume tertiary referral center.

## 2. Materials and Methods

Co-authors discussed key principles of aesthetic optimization in DIEP flap breast reconstruction spanning the full reconstructive continuum, from pre-operative planning through to secondary revision procedures. Consensus was reached regarding an aesthetic framework consisting of key topics including patient selection and pre-operative planning, mastectomy pattern selection, flap design and inset, management of ptosis and nipple position, adjunctive revision procedures, fat grafting, donor site aesthetics, and evolving regenerative and reconstructive adjuncts.

Following identification of these core focus areas, consensus was reached regarding the senior authors’ (CK, DWM, and JW) preferred approaches, technical philosophies, and evidence-informed recommendations based on their collective experience at a high-volume microsurgical breast reconstruction center. This experience encompasses thousands of autologous tissue breast reconstruction procedures and has informed many of the practical considerations discussed throughout the manuscript. In parallel, a targeted literature search was performed using PubMed, Embase, and Web of Science databases to identify studies evaluating aesthetic outcomes, operative techniques, innovations, patient-reported outcomes, and evolving reconstructive strategies in DIEP flap breast reconstruction. Particular emphasis was placed on the literature investigating approaches intended to optimize symmetry, contour, abdominal donor site aesthetics, patient satisfaction, and the broader reconstructive experience. The resulting literature was synthesized alongside our senior authors’ consensus-based recommendations into a comprehensive narrative review focused on modern concepts and emerging strategies to maximize aesthetic outcomes in DIEP flap breast reconstruction.

## 3. Discussion

### 3.1. Aesthetic Considerations in DIEP Flap Reconstruction: Supporting Evidence

#### 3.1.1. DIEP Evolution and Global Aesthetic Considerations

Given that vascular anatomy and microsurgery are the foundation of autologous breast reconstruction, the popularity of autologous reconstruction was catalyzed by research describing the relevant breast anatomy. In 1987, Taylor et al. published a landmark study describing the vascular territories of the human body, which included the major perforators supplying the breasts, as well as the hypovascular and hypervascular planes of the mammary region [22]. The progression of autologous breast reconstruction since this time has focused mainly on utilizing donor sites that provide sufficient volume, minimizing donor site morbidity, and promoting a natural appearance with donor site scars that can be concealed. Latissimus dorsi (LD) flaps were among the first described in autologous breast reconstruction dating back to the mid-1970s [23]. Around this time, the use of the abdominally-based transverse rectus abdominis myocutaneous (TRAM) flap was first described [24,25,26]. Soon the pedicled TRAM flap, followed by the free TRAM flap, became the gold standard [27]. TRAM flaps, however, were often associated with significant donor site morbidity due to the extensive removal of the rectus abdominis muscle. This problem led to one of the latest refinements, the DIEP free flap, which retains the advantages of the TRAM flap but maintains abdominal wall integrity and strength. The DIEP flap has thus mostly replaced the TRAM flap in autologous breast reconstruction today [28].

The breast reconstruction process begins with diagnosis either of a high-risk genotype or active breast cancer, followed by mastectomy and eventually reconstruction. Achieving an aesthetic outcome does not begin at the reconstruction stage; rather, it begins when operative plans are first discussed with the patient. There are several areas of variability in a patient’s surgical path before final reconstruction that may contribute to the final DIEP flap outcome. These points of consideration in the treatment path include, but are not limited to, the following: (1) planning for adjuvant radiation therapy, (2) mastectomy incision design and management of the nipple-areolar complex (NAC), and (3) reconstructive sequencing along with adjunctive procedures. It is imperative that each of these points, among others, are addressed and planned through patient counseling and education, eliciting patients’ priorities and desired outcomes, and shared decision-making as early as possible in their operative treatment path.

#### 3.1.2. Radiation Therapy: Approaches to Maximizing Aesthetic Outcomes

Radiation therapy for breast cancer plays an important role in treatment and is indicated based on several factors, such as nodal involvement, tumor size, cancer stage, and surgical margins. While effective as part of oncologic treatment, radiation to reconstructed breasts is associated with increased rates of adverse outcomes [29]. Several studies have demonstrated that irradiation of the DIEP flap results in increased complications including fibrosis, fat necrosis, volume loss, contour irregularities, contracture, movement restriction, and flap failure [30,31].

Historically, the timing of postmastectomy radiation therapy relative to autologous breast reconstruction has been a topic of debate. Immediate autologous tissue breast reconstruction, in which the flap is exposed to postoperative radiation, is associated with higher rates of late complications compared with delayed reconstruction, where autologous tissue breast reconstruction is performed after completion of radiation therapy [32,33]. Although immediate breast reconstruction confers advantages including reduced total treatment time and number of surgeries, many plastic surgeons have shifted to delayed reconstruction [34]. Postmastectomy radiation therapy is generally considered a relative contraindication to immediate autologous breast reconstruction due to the increased risk of complications, including those reported by our own authors [35,36,37]. The current data and our own institutional experience support delaying DIEP flap breast reconstruction until after radiation therapy is completed. This is commonly achieved through a delayed reconstructive approach, in which mastectomy is performed with an initial temporizing implant-based reconstruction (using a tissue expander or implant), followed by adjuvant radiation therapy and subsequent DIEP flap reconstruction months later [31]. This approach, termed delayed-immediate reconstruction, is supported in these cases to mitigate the adverse effects of radiation on the autologous flap by maintaining the breast envelope with a tissue expander or an implant following mastectomy [38,39,40]. Multiple studies have demonstrated that this approach is associated with more favorable aesthetic outcomes [41,42]. Historically, subpectoral implant-based reconstruction, either with tissue expanders or implants, was preferred. However, prepectoral placement is now increasingly utilized when intraoperative conditions allow, as it preserves native anatomy and decreases morbidity associated with the disruption of native anatomy. This approach is particularly favored in the setting of delayed-immediate autologous breast reconstruction, as definitive autologous reconstruction ultimately requires a prepectoral plane analogous to the native breast. Additionally, when delayed reconstruction is pursued, available data suggest that complication rates are not significantly influenced by the duration of delay prior to autologous breast reconstruction [43]. In a prior study of 303 patients undergoing postmastectomy radiation therapy, Arnautovic et al. found no significant differences in complication rates between patients who underwent autologous breast reconstruction, including DIEP flap (86.2%) and free TRAM flap (8.9%) reconstruction, within one year versus beyond one year following completion of radiation therapy [44]. Interestingly, it was noted that the probability of any major complication peaked 2 to 6 months after radiation therapy completion; thus, the authors concluded that delayed autologous breast reconstruction can be safely performed beginning 6 months after postmastectomy radiation therapy completion. A more recent study evaluating 514 flaps among 339 patients showed no statistically significant differences in reoperations, flap thrombosis, flap failure, infection, wound dehiscence, or operative time when comparing cases with or without postmastectomy radiation therapy [45]. Of note, the postmastectomy radiation therapy group was stratified into cohorts based on the time from completion of radiation to autologous breast reconstruction, including 0–3 months, 3–6 months, 6–12 months, and 12+ months, and found no statistically significant differences when comparing cases at these various time intervals.

#### 3.1.3. Mastectomy Technique

**Figure 1:**
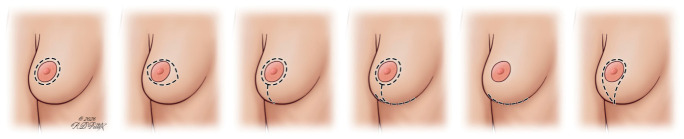
Mastectomy incision pattern illustration, with the dashed lines representing example incision. Common mastectomy incision designs vary in location and extent, influencing both surgical access and preservation of the skin envelope and nipple–areolar complex. These patterns play a critical role in shaping the reconstructive plan and ultimately impact aesthetic outcomes following DIEP flap reconstruction.

Mastectomy technique is among the first surgical considerations when planning for autologous breast reconstruction and has direct implications for subsequent reconstruction and aesthetic outcomes. Options range from radical and total mastectomies to more conservative approaches including skin-sparing (SSM) and nipple-sparing mastectomies (NSM). Over time, breast surgery has increasingly shifted toward tissue-preserving approaches. As aesthetic outcomes have become a greater priority, SSM and NSM have gained widespread adoption with significantly improved patient-reported and aesthetic outcomes [46,47,48]. These techniques optimize preservation of the native breast skin envelope and NAC, thereby facilitating autologous reconstruction while maintaining oncologic safety and local disease control in appropriately selected patients [49]. Several incision patterns can be utilized (Figure 1) depending on the breast surgeon’s comfort level for access and the desired final outcome. It is reassuring that the oncologic safety of SSM and NSM is well established in the literature [41,49,50,51,52,53].

In fact, several governing bodies have previously discussed eligibility criteria for the oncological safety of NSM. The US National Comprehensive Cancer Network (NCCN) 2026 guidelines propose several criteria for NSM candidacy. Patients undergoing mastectomy for early-stage cancer, ductal carcinoma in situ, prophylaxis, and those with no NAC involvement are candidates. NSM is contraindicated in patients with any nipple or subareolar involvement found on imaging or those with Paget’s disease or bloody discharge associated with malignancy. Furthermore, these guidelines recommend considering breast shape, size, and NAC position. Even though macromastia and/or significant ptosis are not absolute contraindications, guidelines recommend careful patient selection, use of adjunctive reduction or delay techniques to bolster NAC perfusion, and intraoperative assessment of NAC perfusion [54]. NSM has also been proposed as an excellent option for many patients requiring prophylactic mastectomy following identification of BRCA mutations [47]. In addition, recent studies have shown NSM to be safe and effective in older patients (older than 65 years) [55]. It is not surprising that preservation of the NAC is associated with significantly improved cosmetic outcomes and patient satisfaction, including psychosocial (*p* = 0.01) and sexual well-being (*p* = 0.02) BREAST-Q domains compared to SSM patients [56]. Therefore, when appropriate, NSM should be the preferred option and strongly considered.

#### 3.1.4. Management of Ptotic Breasts

Surgical planning becomes more complex in patients with large ptotic breasts. Traditionally, and even today in many centers, NSM is not available to patients with severe macromastia or moderate-to-severe breast ptosis due to concerns about increased risk of NAC necrosis and significant asymmetry. The literature has consistently illustrated that surgical complications such as mastectomy skin flap necrosis or NAC necrosis are increased in such patients due to the heightened perfusion demands [57,58,59,60,61]. These complications have a significant impact on the aesthetic outcomes of DIEP flap reconstruction since they can lead to asymmetry and poor cosmesis that can ultimately become too challenging to revise. It is thus not surprising that previously reported outcomes from the American Society of Breast Surgeons Nipple Sparing Mastectomy Registry showed that only 15% of NSMs are performed in patients with grade II and only 3.9% in patients with grade III ptosis [62].

Another challenging aspect of reconstruction in patients with macromastia and/or ptotic breasts is skin envelope tailoring after mastectomy [63]. As discussed by Nahabet et al., even though Wise pattern incision techniques lead to the most aesthetically pleasing outcomes due to effective manipulation of the skin envelope that provides desirable breast projection and shape, their unacceptably high rates of mastectomy skin flap necrosis and NAC necrosis have made them less attractive. On the other hand, circumvertical pattern incision techniques are often limited in their ability to reduce the skin envelope sufficiently but have a more favorable complication profile [64].

For many of the reasons mentioned above, NSM was traditionally contraindicated in patients with macromastia and moderate-to-severe ptosis. While ptotic breasts may present challenges, the recent literature supports several safe and effective approaches to complete a mastectomy and reconstruction in these patients [64]. Approaches to autologous breast reconstruction in patients with ptotic breasts can broadly be categorized as either single-stage or two-stage strategies. Single-stage approaches can involve NSM with a pedicled NAC or SSM with NAC grafting. Two-stage approaches have greater diversity; they may involve nipple delay, pre-mastectomy mastopexy, or postmastectomy mastopexy. The literature reflects this variety of techniques, but remains unclear as to whether staged or immediate autologous breast reconstruction is the preferable approach [57,65]. Moreover, cost is an important consideration in the selection of reconstructive timing. For instance, delayed reconstruction procedures are associated with increased upfront costs given the increased number of operations [66]. Cost has also been cited as a major disadvantage of staging mastopexy, as in the case of nipple delay procedures [67].

A single-stage approach including simultaneous NSM, reduction mastopexy, and DIEP flap reconstruction has been performed in severely ptotic breasts with favorable outcomes [68]. However, this simultaneous single-stage approach may face a heightened risk of NAC ischemia and be limited by how much the NAC can be mobilized given the need for maintaining dermal continuity [69,70]. Pre-operative nipple delay prior to mastectomy and reconstruction has also been shown to potentially offer a protective benefit against NAC necrosis by facilitating adaptive changes in NAC blood supply in patients at higher risk, such as those with macromastia or advanced ptosis [71].

Adjunctive mastopexy has been shown to be oncologically safe regardless of timing [58,72,73,74]. Satisfactory outcomes have been attained across a wide range of approaches, with mastopexy performed almost two years before or as long as 185 days after mastectomy and reconstruction [58,75]. Momeni and colleagues performed the largest retrospective analysis to date evaluating the outcomes of women with grade II or III ptosis and/or macromastia who underwent bilateral (oncoplastic) reduction/mastopexy followed by bilateral NSMs and immediate autologous breast reconstruction, demonstrating the safety of this approach [57]. A different group from New Orleans, DellaCroce et al., completed one of the largest series to date evaluating patients with grade II/III ptosis who underwent NSM with immediate perforator flap reconstruction and subsequently underwent a mastopexy procedure as a second stage. The authors demonstrated that full mastopexy, including a complete full-thickness periareolar incision and nipple–areola complex repositioning on the breast mound, can be safely performed because the underlying flap provides adequate vascular ingrowth to support the perfusion of the NAC despite complete incisional interruption of the surrounding cutaneous blood supply [75].

**Figure 2:**
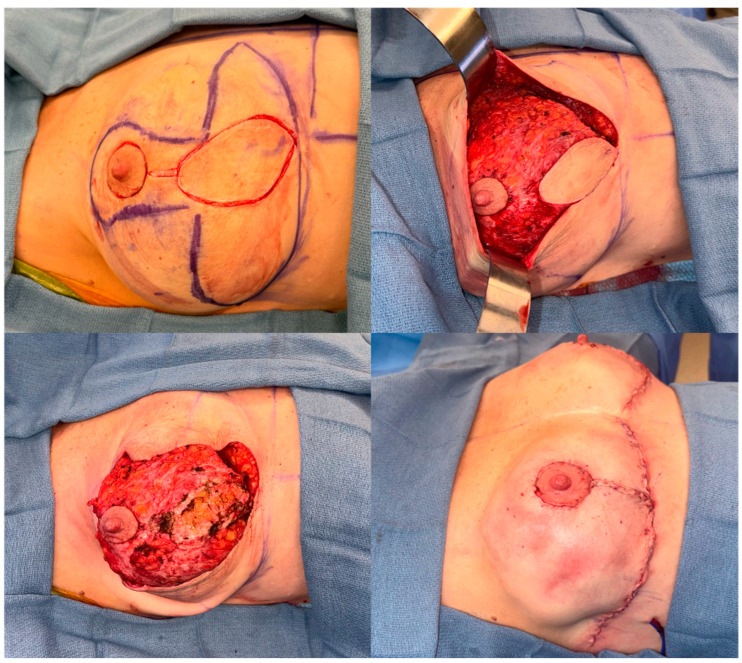
Intraoperative photographs showing a bilateral Wise pattern mastopexy to correct NAC positioning in a patient who had previously undergone bilateral DIEP flap reconstruction.

In our authors’ opinion, the decision to pursue one approach over another is highly dependent on an individual patient’s anatomy and desires, as well as a few other parameters such as the possible need for postmastectomy radiation therapy; there is no ‘one size fits all’. In the senior authors’ experience, there are situations in which the two-stage approach with initial tissue expander placement has consistently been advantageous over a single-stage approach, such as when the estimated flap weight exceeds approximately 600–700 g. Similarly, we have found the two-stage postmastectomy mastopexy approach to be among the most versatile in managing significant ptosis or macromastia. The robust NAC revascularization from the underlying well-perfused autologous flap is adequate to maintain the viability of the NAC when the surrounding contributions of the skin are disrupted with full-thickness incisions; therefore, it is possible to completely reposition the preserved NAC in a manner similar to a routine mastopexy with any of the typical skin resection patterns (Figure 2). This allows for modification of the breast shape, size, and NAC position during the second stage most of the time without any restrictions.

#### 3.1.5. Free NAC Grafting and Nipple Reconstruction

Many patients are still not appropriate candidates for NSMs for various reasons, such as the malignancy being too close to the NAC [63]. Free nipple grafting has become a suitable option for such patients. Egan et al. illustrated that free nipple grafts led to similar outcomes and overall aesthetic satisfaction rates compared to local flap reconstruction, despite all patients experiencing partial graft hypopigmentation as expected, which did not affect overall outcomes [76]. Other authors have reported similar findings with overall high satisfaction rates with free nipple grafting [77]. Intraoperatively, frozen sections of deeper tissue from the harvested full-thickness NAC is sent to pathology to ensure all malignant tissue has been excised [76,78]. After appropriate thinning and defatting of the NAC, it is inset to the desired position over the deepithelialized part of the newly reconstructed breast [76]. One study reported that outcomes following free nipple grafting were generally comparable to those of local flap NAC reconstruction. For example, projection was maintained in 54% of grafts, and overall NAC aesthetics were rated at 3.7 ± 0.7 on a 5-point scale [76]. The benefits of free nipple grafting include utilizing the patient’s native NAC, elimination of the need for maintenance tattooing and secondary procedures, as well as cost and psychosocial benefits.

Nipple reconstruction is another option, most commonly performed as a delayed procedure following autologous breast reconstruction; however, increasing evidence suggests benefits associated with a proactive approach rather than treating nipple reconstruction as a secondary procedure [79,80]. In cases where nipple preservation is not possible, patients can still undergo eventual nipple reconstruction with local flap options, such as the C-V flap, with subsequent tattooing several months later since any type of nipple reconstruction leads to higher patient satisfaction [13,81,82,83]. Indeed, nipple reconstruction has been shown to significantly improve general and aesthetic satisfaction scores (*p* < 0.0001), and some authors have described methods of modified Wise pattern SSM with autologous breast reconstruction to facilitate simultaneous local flap nipple reconstruction suitable in ptotic breasts [84,85]. This approach allows these procedures to be performed during the primary reconstruction; as such, they need not be considered as exclusively secondary, complementary procedures.

Tattooing has also emerged as an increasingly popular option for NAC reconstruction, with one recent 2025 study finding that rates of tattoo-only NAC reconstruction have significantly increased over the 15-year study period (β = 0.173, *p* < 0.0001) [86]. Tattoo-based reconstruction is an excellent option for patients wanting to avoid further invasive surgery or for patients without adequate native tissue for a local flap-based reconstruction. Tattooing, particularly 3-dimensional NAC tattoo reconstruction, has been shown to significantly improve BREAST-Q scores following breast reconstruction, with color fading being the most common reason for tattoo revision [87,88,89].

#### 3.1.6. Neurotization and Reinnervation: Maintaining Sensation Following Breast Reconstruction

**Figure 3:**
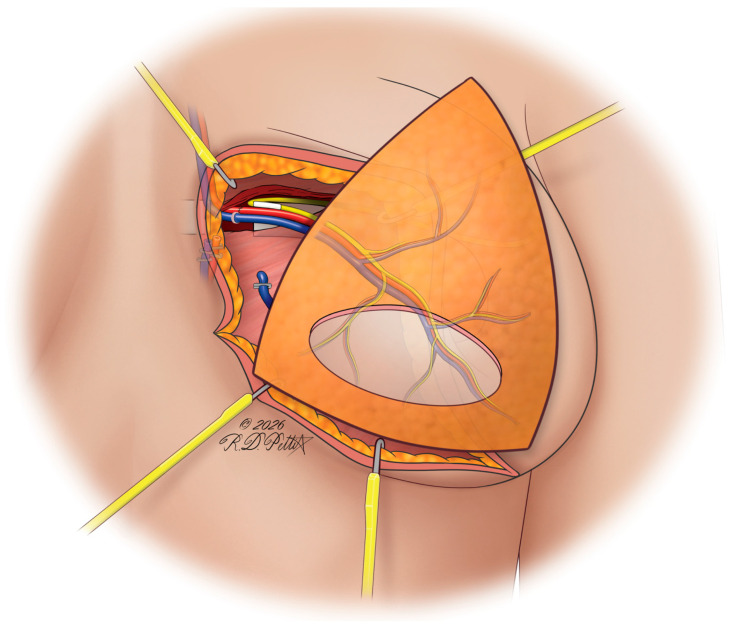
Illustration of nerve allograft for neurotization for DIEP flap coaptation. Microsurgical coaptation using interposition nerve allografts enables restoration of sensation by bridging donor and recipient intercostal nerves when direct repair is not feasible. This approach facilitates more reliable and earlier sensory recovery across the reconstructed DIEP flap, enhancing functional and aesthetic outcomes. The allograft bridges the gap when a tension-free direct repair is not achievable.

Among the many factors known to influence patient quality of life after mastectomy and breast reconstruction, impaired sensation of the remaining skin and NAC is known to have a potentially debilitating impact on patient satisfaction. Maintaining or restoring sensation in DIEP flap reconstruction is crucial for several reasons; patients are at increased risk of injury due to reduced sensation, sensation of the breasts plays a role in sexual function, and patients identify better with their reconstructed breasts if they retain sensation to touch [90]. Rates of retained sensory function of the breast skin and NAC following mastectomy and reconstruction are reported to range between 0% and 47%, contributing to patient dissatisfaction [91]. Without taking steps to specifically promote innervation, sensation after DIEP flap reconstruction returns only through peripheral regeneration, which is often slow and incomplete. Neurotization refers to the reinnervation of the autologous flap through microsurgical coaptation of the intercostal nerves, allowing more rapid and robust recovery of sensation than passive nerve regeneration alone. Microsurgical nerve coaptation has uniformly been shown to yield significant improvements in sensation, facilitating earlier and superior breast sensibility that is more uniformly distributed [92,93,94,95]. The use of nerve allografts is indicated when direct end-to-end nerve coaptation is not possible and has been shown to yield comparable long-term breast cutaneous sensitivity and subjective patient-reported outcome measures (Figure 3) [96].

The selection of donor and recipient nerves is also a current topic of investigation. Typically, DIEP flap coaptation is performed with coaptation of a donor T11 or T12 intercostal nerve sensory branch to a recipient anterior cutaneous branch of T3, though use of the lateral cutaneous branch has been described [97]. For donor nerves, current evidence supports the use of T12 intercostal nerve sensory branches because they are associated with superior cutaneous sensitivity across most breast regions throughout recovery when compared with T11-innervated breasts [98]. Similarly, use of the intercostal anterior cutaneous branches as the recipient nerves appears to promote superior sensory outcomes and patient satisfaction when compared to the lateral cutaneous branches [99].

#### 3.1.7. Operative Sequencing: Immediate and Delayed DIEP Flap Reconstruction

Outside the context of radiation therapy, the decision to pursue mastectomy with immediate autologous breast reconstruction or to delay autologous breast reconstruction with or without the use of tissue expanders represents a key determinant of aesthetic outcomes in DIEP flap reconstruction. Both approaches are generally regarded as oncologically safe [100]. Generally, many surgeons advocate for delayed reconstruction following mastectomy, involving the immediate placement of tissue expanders, as it allows the skin envelope and breast footprint to be preserved and optimized over time, facilitates controlled pocket formation, and ultimately supports improved contour, symmetry, and long-term aesthetic outcomes. With respect to postoperative complications, one multicenter study conducted by Beugels et al. showed that among 397 immediate and 513 delayed DIEP flap reconstructions, hematoma formation (OR 2.91, 95% CI 1.59–5.30, *p* = 0.001) and seroma formation (OR 3.60; 95% CI 1.14–11.4, *p* = 0.029) were more common in immediate reconstruction [101]. Accordingly, a 2022 meta-analysis of 14,034 patients showed that immediate reconstruction patients are more likely to experience infection (OR 1.41, 95% 1.04–1.92, *p* = 0.03), hematoma and seroma formation (OR 2.01, 95% CI 1.27–3.17, *p* = 0.003), as well as an increase in overall surgical complications (OR 1.30, 95% CI 1.03–1.65, *p* = 0.03) [102]. Several other studies have demonstrated similar outcomes [103,104]. Our authors’ experience is that while immediate reconstruction may confer advantages such as reduced total operative time and more rapid restoration of permanent breast form, it is only preferred in our practice if the harvested flap volume is less than 600–700 g, the breast pocket is large enough, and the skin envelope lax enough to accommodate the flap without exerting undue tension. In most other circumstances, the authors prefer delayed-immediate reconstruction or delayed reconstruction if adjuvant radiation is needed.

### 3.2. Maximizing Cosmesis: Execution and Technique

#### 3.2.1. Pre-Operative Perforator Selection and Marking

Pre-operative planning is a crucial step in optimizing aesthetic outcomes in DIEP flap breast reconstruction. The surgeon’s meticulous attention to detail and individualized surgical plan in this phase can often make the difference between acceptable and optimal outcomes. Important considerations during this phase include pre-operative imaging for perforator mapping and pre-operative marking to maximize aesthetic results by taking into account restoration of the breast footprint, management of the skin envelope, inframammary fold definition, and lateral breast definition. These considerations can have significant variations between immediate and delayed reconstruction and even unilateral versus bilateral reconstruction. Regardless of timing or type of reconstruction, pre-operative mapping and selection of the dominant perforator or the most suitable perforators that will allow expedited dissection with the least amount of donor site morbidity has become standard practice when planning DIEP flap breast reconstruction in most institutions. The appropriate selection of perforator(s) can prevent undesirable outcomes, such as bulges or hernias at the donor site and fat necrosis at the recipient site, which can subsequently lead to more optimal aesthetic outcomes [105,106]. Many imaging modalities exist that help map out the abdominal wall vasculature, such as duplex ultrasound (US), contrast-enhanced ultrasound (CEUS), computed tomography angiogram (CTA), and magnetic resonance angiography (MRA). Pre-operative CTA imaging has become the gold standard due to its benefits over other imaging modalities. It is more cost-effective and user-friendly than MRA and has proven superior in delineating the caliber, location, and course of the musculocutaneous perforators compared to duplex US and Doppler [107,108,109]. Many studies have shown that pre-operative imaging prior to DIEP flaps can decrease complication rates and operative time [110,111,112,113,114,115,116,117,118]. Therefore, pre-operative CTA imaging can theoretically lead to more optimal aesthetic outcomes, although studies investigating this potential link are currently lacking. Despite the growing utility in diverse surgical contexts, CTA is also associated with additional burdens including elevated costs, radiation exposure, and contrast exposure [119]. While some studies argue that including CTA in DIEP flap planning is cost-neutral or may even save costs by reducing complication rates, future research is necessary to characterize its benefits [116].

More recently, innovations in pre-operative planning have included augmented reality (AR)-based systems and virtual reality (VR)-based systems driven by CTA imaging. The system utilizes machine learning to create a map of a vessel’s course and maximize perforator visualization, which can improve accuracy and reduce complication rates by better familiarizing surgeons with a patient’s vascular anatomy [120]. Similarly, technologies used for virtual surgical planning common in orthognathic surgery or neurosurgery have been described in DIEP flap planning. One recent systematic review has shown that AR-based and VR-based planning and perforator mapping significantly reduced operative time and, as a result, cost savings have been described in both AR-based and VR-based systems, though this does not account for the associated learning curve [121]. AR has been used for intraoperative navigation in DIEP flap reconstruction as well, showing promise in guiding fascial dissection for efficient perforator harvest [122]. This combination of cutting-edge imaging and artificial intelligence technologies could, therefore, theoretically improve aesthetic outcomes by maximizing accuracy, though further research is needed to characterize this relationship, as well as the expected cumulative cost.

Pre-operative marking is a fundamental step in optimizing aesthetic outcomes in DIEP reconstruction. This serves as the blueprint for the surgeon, and it can vary between immediate and delayed reconstruction. Nevertheless, there are some general principles that are applicable regardless of the timing of reconstruction. With the patient in a standing position, the DIEP flap is commonly outlined pre-operatively as an elliptical or boat-shaped design on the abdomen, typically connecting anatomical landmarks, such as the bilateral anterior superior iliac spines (ASIS), umbilicus, and mons pubis, with additional markings for perforators [123]. There have been other studies that utilized individualized templates via CTA for pre-operative marking, but these lacked a methodological approach to optimize donor site aesthetics [124]. Xue et al. proposed a step-by-step approach to DIEP flap pre-operative marking by identifying some key anatomic landmarks, such as the bilateral ASIS, umbilicus, sternal notch, and vaginal cleft, in order to produce more reproducible, reliable, and aesthetic donor site outcomes through a standardized approach [125].

Our authors’ recommendation is to try to keep the upper abdominal incision as low as possible. In our experience, doing so allows the final scar to be in a more ideal position, low along the lower abdomen, which is key for concealment and for the overall aesthetic outcome of the donor site. Of course, we always ensure that the flap design incorporates the desired perforators that will be harvested. For the recipient site, pre-operative marking includes the suprasternal notch, the chest midline, the breast footprint, the desired inframammary fold, and the mastectomy incision lines and the desired access point for the breast pockets [21,38,126,127]. In addition, the chest width is often measured to ensure appropriate flap width [13]. The overarching goal is to create a breast mound that is symmetrical in volume, projection, and shape to the contralateral breast in unilateral cases or, overall, aesthetically pleasing and symmetric in bilateral cases.

#### 3.2.2. Technique and Execution: Delayed vs. Immediate Reconstruction

Appropriate marking in this setting can lead to desirable integration of the DIEP flap with the postmastectomy skin envelope to achieve symmetrical volume and projection. In addition, the quality of the mastectomy skin flap has a significant impact not only on the success of the reconstruction but also on the aesthetic results [59,63,128,129]. This holds true for both SSM and NSM approaches. Incision pattern, dissection technique, and flap thickness and perfusion are important factors affecting aesthetic outcomes [59,63,128]. There are many incisional options for both mastectomy approaches (Figure 1). In nipple-sparing immediate DIEP flap reconstruction, there are many studies that have found that the periareolar incision carries high risk for NAC necrosis, with most of the literature showing lower complication rates and NAC ischemic injury with inframammary fold incisions compared to periareolar and Wise pattern incisions [130,131,132,133,134,135,136,137]. More recent studies have shown that inframammary or inverted T incisions were associated with the highest rates of ischemic injury compared to vertical and radial lateral incisions, likely due to the increased traction required for reaching recipient vessels in inframammary incisions and the disruption of overall perfusion due to the more extensive incisions in inverted T incisions [138]. Understanding the patient’s anatomy and tailoring the incision pattern to their reconstructive and aesthetic goals is important, while also educating them about the risks involved with each incisional option. Dissection technique is mainly dependent on surgical skill set, but preserving the superficial subcutaneous tissue to avoid ischemia is of utmost importance [59,139]. Preserving as many of the medial intercostal perforators is also key to the vascularity of the mastectomy skin flaps. A patient’s body mass index and breast habitus affect the overall flap thickness, which should also be considered in every case [59].

**Figure 4:**
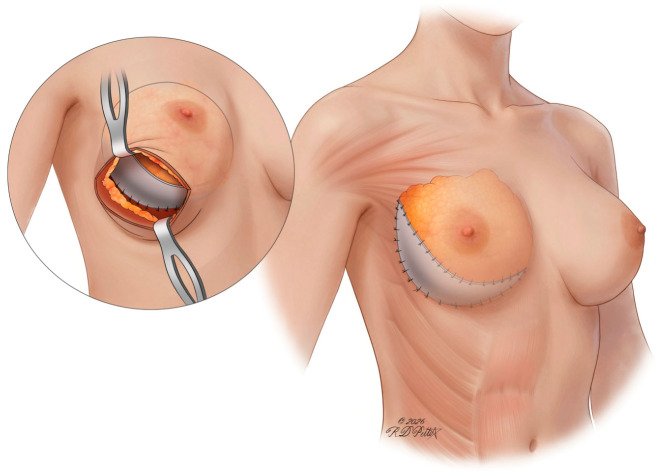
Illustration of mesh to re-establish the inferolateral breast borders. Placement of a biosynthetic mesh sling along the inferior and lateral aspects of the breast helps recreate the native footprint and define the inframammary fold. This provides structural support for the DIEP flap, improving contour, projection, and long-term stability of the reconstruction.

In immediate reconstruction, the reconstruction is performed concurrently with the mastectomy, and as such it is imperative for the plastic surgeon to communicate effectively with the breast surgeon and design the appropriate markings in anticipation of the postmastectomy defect. In addition, it is important to recognize how much the normal boundaries of the breast footprint have been disrupted after the mastectomy [21,140]. The pre-operative markings of those boundaries need to be examined, and if the surgeon deems it necessary, these need to be restored with re-suturing to the chest wall to re-establish the footprint with the appropriate height and width on the chest wall [21]. The inframammary fold and the lateral border of the footprint are the most commonly violated borders and, as such, particular attention must be paid to the inframammary fold to ensure an aesthetically pleasing outcome [21]. Correcting and reinforcing the inframammary fold and the lateral border at the time of mastectomy is crucial for optimizing symmetry and overall cosmesis. This can be accomplished by suturing the mastectomy flaps down to the chest wall with caution to avoid inadvertently injuring the lateral intercostal nerves that can be found at the edge of the pectoralis muscle [39]. If the surgeon feels that the sutures may not adequately restore the inferior and lateral breast borders, a bioprosthetic mesh can also be utilized to help re-establish the inferolateral breast borders (Figure 4). Furthermore, careful pre-operative marking of any discrepancies between the heights of the inframammary fold is crucial in improving overall final symmetry [39]. Achieving symmetry in unilateral and bilateral immediate DIEP reconstruction is one of the most fundamental aesthetic goals, and yet it can often be challenging. In unilateral reconstruction, the contralateral breast is used as the template, and specific measurements are taken to guide the pre-operative markings and ultimate reconstruction of the breast [21]. In bilateral reconstruction, ensuring symmetry of the pockets is crucial, and it starts with the breast surgeons when they perform the mastectomy, and then is fine-tuned by the plastic surgeons with any necessary further excisions and adjustments. Another important aesthetic consideration in immediate DIEP reconstruction pertains to the appropriate adjustments to the skin envelope. When there is excess skin, meticulous excision can ensure a more aesthetic integration of the DIEP flap.

**Figure 5:**
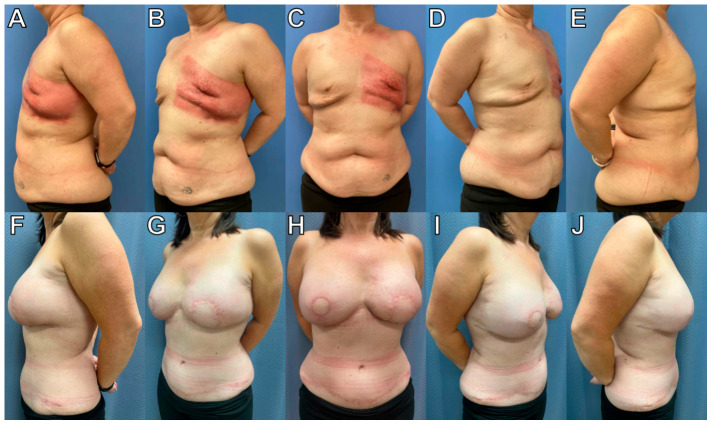
A 48-year-old patient with left breast cancer underwent bilateral mastectomy with sentinel lymph node biopsy followed by adjuvant chemotherapy and radiation. Delayed bilateral DIEP flap reconstruction demonstrates restoration of breast contour in the radiated chest. (**A**–**E**) Pre-operative photographs. (**F**–**J**) Postoperative photographs.

Delayed DIEP reconstruction presents with its own challenges. Many of these patients undergo adjuvant radiation after their mastectomies prior to autologous breast reconstruction, which can result in a poor skin envelope with fibrotic changes, tissue contraction, fat necrosis, and overall distortion of the breast footprint [21,39]. The surgeon must pay attention to any scarred mastectomy skin tissue that has lost its natural pliability due to radiation and formulate an appropriate plan for excision. This is particularly important during the evaluation of radiated skin along the inferior pole of the breast and centrally, which often requires excision to produce a more aesthetically pleasing lower pole during flap inset [39]. However, there are also times where excision of the upper pole skin or transection and scoring of constricting bands to release the skin from the inside are necessary to accommodate the flap and to shape the upper pole [21]. It is also common to encounter excessive scarring on the lateral skin envelope towards the axilla, which limits contouring of the upper pole when the flap is inset. Some authors ensure sufficient lateral skin laxity by back-cutting the radiated skin towards the axilla, which can help recruit skin that has naturally migrated towards the axilla after mastectomy, leading to a more aesthetically pleasing upper pole [13,21,39]. The authors also stress the importance of preserving the lateral breast skin envelope but undermining cephalad to minimize the risk of step-offs. Accordingly, removal of radiated tissue has many benefits, including ptosis improvement, excision of damaged skin, better contouring of the lower pole, and improved overall symmetry to the contralateral breast (Figure 5) [13]. As mentioned previously, it is important to design a DIEP flap that is wider than the chest width to ensure adequate skin coverage, especially when planning lower pole skin resection [13].

Recreating the desired new inframammary fold by marking the appropriate location with the patient in a standing position is another crucial pre-operative consideration. In unilateral cases, the goal is to create a symmetric inframammary fold to the contralateral side, while in bilateral cases the goal is to recreate a well-defined inframammary fold location bilaterally with overall symmetry. The surgeon needs to consider all the factors that displace the inframammary fold inferiorly. Such factors include pulling forces from the abdominal closure and radiated tissue expander [39]. Therefore, surgeons have historically advised marking the inframammary fold higher than anticipated because revisions of folds that are positioned low are much more challenging [13,21,39]. In unilateral cases, Blondeel et al. recommended placing the new inframammary fold 2 to 3 cm higher than the contralateral non-reconstructed breast IMF, and even slightly higher if there is significant fibrotic radiated tissue requiring excision [21]. Sinik et al. recommended leaving a 1 cm skin cuff along the desired inframammary fold when excising lower pole skin for flap inset to recreate a more aesthetic inframammary fold after flap inset [13]. Lastly, performing complete capsulectomy with removal of acellular dermal matrix is an important intraoperative consideration in delayed-immediate reconstruction after removing the tissue expander or implant. This facilitates appropriate expansion and contouring of the mastectomy pocket with a more aesthetic integration of the DIEP flap and better overall symmetry. After complete capsulectomy, the skin’s increased pliability optimizes flap shaping and skin envelope re-draping [13]. All these enable the plastic surgeon to optimize the contour and symmetry of the new breast without the limitations or anatomical distortions that would result from a residual capsule or acellular dermal matrix.

#### 3.2.3. Flap Shaping

Sculpting DIEP flaps into three-dimensional aesthetically pleasing breasts remains one of the most challenging aspects of autologous breast reconstruction. DIEP flaps typically adopt a triangular shape with poor projection upon harvest due to the inherent anatomy and contour of the lower abdomen [141,142]. Ultimately, the shape of the new breast mound relies on the artistic manipulation of the layers of DIEP flaps, including the skin and the subcutaneous fat/Scarpa’s fascia [141,142]. Historically, surgeons had primarily focused on DIEP flap insetting techniques as the principal method of sculpting the new breast. Even though insetting remains an important component of breast contouring, over the last decade, novel techniques in flap pre-shaping have introduced innovative ways to optimize aesthetic outcomes. In unilateral breast reconstruction with DIEP flaps, such advancements took advantage of the growing capabilities of 3D photography in order to create a breast that is symmetric to the contralateral non-reconstructed breast. Tomita et al. described their method of utilizing individualized 3D-printed molds based on pre-operative 3D photography of the contralateral breast. The DIEP flaps were placed in these molds intraoperatively, and appropriate adjustments were made. They reported satisfactory cosmetic outcomes based on postoperative volumetric measurements via 3D imaging [143]. Chae et al. expanded on this 3D-printer technology by introducing their coning technique, which involved placing concentric purse-string sutures within the sub-Scarpa’s fat of the DIEP flap to enhance projection. They utilized a 3D-printed mirror image of the contralateral breast intraoperatively to guide the desired projection and extent of coning. The small sample size of this study (three patients) along with questions raised about the potential higher risk of pedicle compromise due to the close proximity of the concentric sutures on the underside of the flap have prevented this technique from being adopted [144].

**Figure 6:**
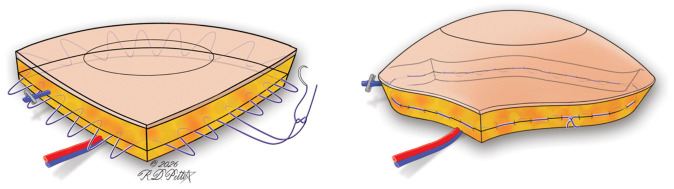
Illustration of DIEPplasty purse-string technique. A circumferential purse-string suture placed within the Scarpa’s fascia is used to conically shape the DIEP flap, enhancing central projection and defining the breast mound. This technique allows for controlled, uniform tension distribution to achieve a more natural contour and improved aesthetic outcomes. Distributing tension circumferentially, rather than at discrete points, is what generates a more controlled central projection.

Odobescu et al. proposed a novel technique of shaping the flap by utilizing two 2-0 polydioxanone purse-string sutures in the Scarpa’s fascia, which start at the 10 and 2 o’clock positions and are tied at the 6 o’clock position to the desired tension to evenly distribute the effect of the purse-string to the periphery of the flap. The authors suggested that this technique is simple and yet reproducible, safe, and effective in optimizing the shape and projection of the desired breast [145]. We introduced another innovative technique, the purse-string DIEPplasty, which utilizes one 0 polydioxanone continuous purse-string suture through the Scarpa’s fascia [142]. This technique has been adopted by our authors since March of 2023 with promising long-term results [142]. Unlike the two purse-string sutures, the single purse-string DIEPplasty provides a more homogenous distribution of tension throughout the flap. It shapes the DIEP flap analogously to an implant with a central dome-like projection and round base, which optimizes the aesthetics of the newly reconstructed breast mound (Figure 6) [142]. These advancements in flap shaping prior to insetting exemplify the artistic curiosity and technical inquisitiveness that drive reconstructive plastic surgeons.

#### 3.2.4. Flap Insetting

**Figure 7:**
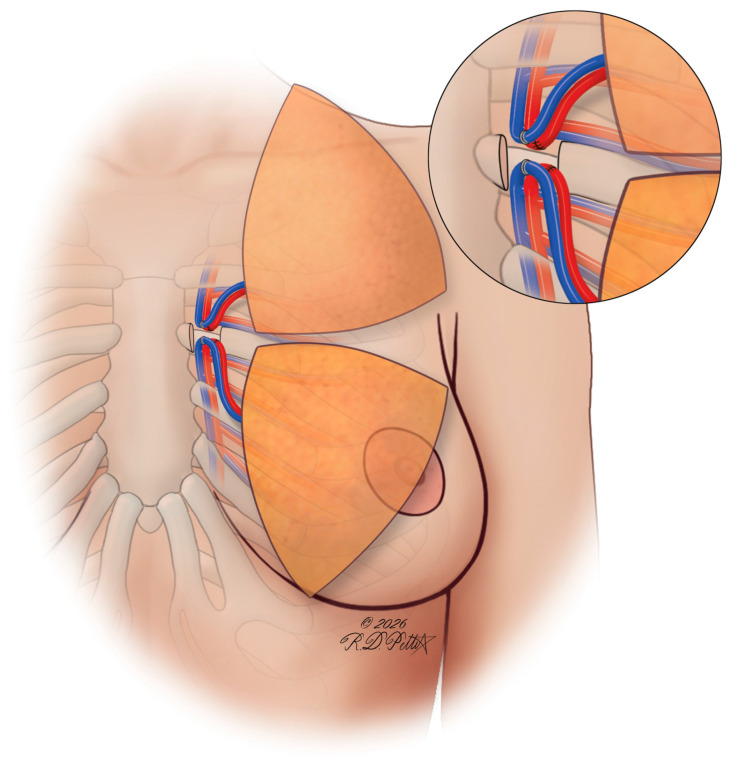
Illustration of stacked or conjoined flaps. Stacked or conjoined DIEP flaps utilize multiple vascular pedicles to augment volume and improve contour in patients with insufficient single-flap tissue. This approach enhances projection and allows more precise shaping of the reconstructed breast while maintaining reliable perfusion.

**Figure 8:**
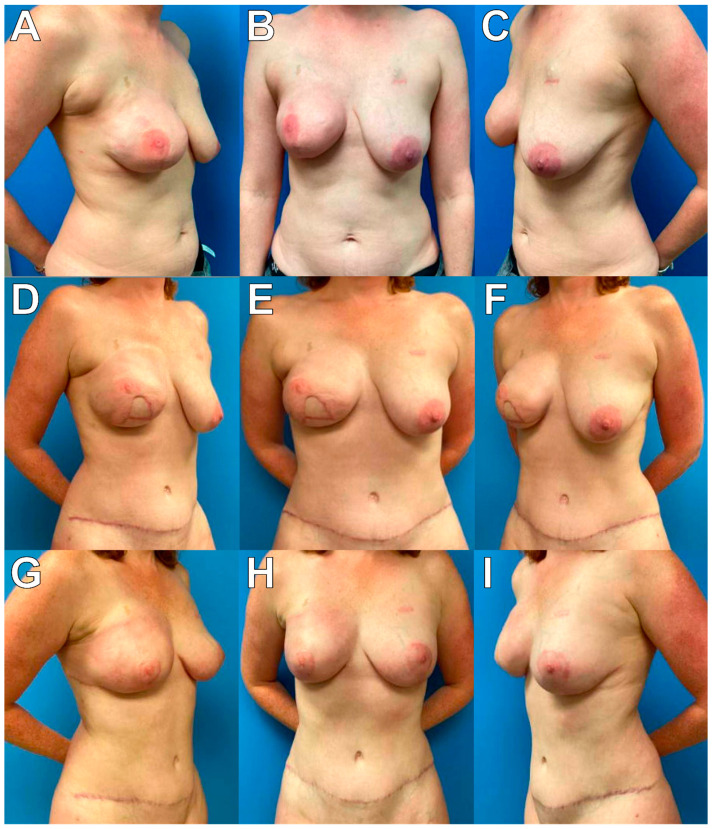
Preoperative and postoperative photographs of a delayed DIEP flap that has undergone radiation. A 40-year-old patient with right breast cancer underwent right SSM with immediate breast reconstruction with tissue expander placement followed by adjuvant radiation therapy. Delayed bilateral stacked DIEP flap reconstruction, and subsequent revision mastopexy and fat grafting demonstrate restoration of breast contour in the radiated chest. (**A**–**C**) Pre-operative photographs. (**D**–**F**) Postoperative photographs following the stacked DIEP flap reconstruction of the right breast. (**G**–**I**) Postoperative photographs following revision, mastopexy, and fat grafting.

When insetting the DIEP flap to the recipient breast, there is an overall preference for choosing the contralateral hemiabdomen and rotating the flap 90 degrees. This has many benefits, including simpler microsurgery, easier access to the superficial inferior epigastric vein if deemed necessary to improve venous outflow, and more ideal conus projection due to placement of the thick midabdominal region in the lower pole of the breast [21]. After anastomoses to the internal mammary vessels, usually at the level of the 3rd or 4th intercostal space, there are many methods that have been described for securing the flap in the breast pocket and creating the desired shape and projection [38]. In unilateral DIEP reconstruction, having sufficient tissue is usually not a problem since surgeons often cross the midline and use zones 1–3, and sometimes even zone 4 if necessary, to recruit the desired tissue volume [38]. Other excellent choices for augmentation of tissue volume with improved projection and versatile flap positioning include the use of stacked or conjoined flaps (Figure 7 and Figure 8). Conjoined flaps are single flaps that are based on more than one pedicle being perfused by multiple perforasomes, whereas stacked flaps are two or more separate flaps, each with its own pedicle [63]. Several different configurations for insetting have been proposed. Salibian et al. showed that stacked or conjoined flaps had lower risk for fat necrosis and lower rate of contralateral breast symmetrizing reduction compared to non-stacked and non-conjoined flaps in unilateral breast reconstruction [146]. In bilateral DIEP reconstruction, the abdomen is typically hemi-dissected and each hemiabdomen is rotated 90 degrees and inset to the contralateral recipient breast. As such, the lateral edge of the DIEP flap is inset on the superior chest wall, while the inferior edge of the flap is inset along the sternal border [38]. This orientation also helps position the thickest part of the flap in the lower pole and the thinnest part of the flap in the upper aspect of the new breast to provide a more natural tapered upper pole appearance [21].

Blondeel et al. described their suture technique that utilizes three key sutures for shaping the DIEP flap. The first suture is placed in the superolateral edge of the pocket between the Scarpa’s fascia and the pectoralis fascia medial to the lateral pectoralis muscle border, which helps secure the flap under the pectoralis tendon. This key suture helps provide some fullness to the lateral border and redefine the anterior axillary fold. The second suture is placed between the lateral edge of the flap and the lateral inframammary fold with appropriate adjustments in tension to optimize the natural transition of the lateral border of the breast to the chest wall and achieve the desired lazy-S shape. The third key suture is placed at the most medial aspect of the newly created inframammary fold to ensure sufficient natural cleavage [21]. Nahabedian et al. emphasized the importance of securing the flap to the chest wall by employing some key sutures at the medial and lateral edges of the DIEP flap. They also proposed additional key sutures that are placed superomedial, inferomedial, and lateral in the new breast pocket, especially in immediate reconstruction when the original breast footprint is larger than the DIEP flap footprint. To improve projection of the flap in unilateral reconstruction, they also described shaping the flap as a cone or creating a lateral fold, which positions zone 2 under zone 1, with zone 3 being placed along the sternal border [38]. All these suturing techniques can help position the flap appropriately on the chest wall and optimize the shape and contour of the new breast by preventing the flap from migrating laterally, which also maximizes superomedial fullness [21,38,39,147]. The additional sutures described by Nahabedian et al. are typically not required in delayed reconstruction since the pocket has been recreated to fit the flap like a hand-in-glove during inset to optimize the contour and projection of the new breast, especially after tissue expander staging [13,38]. Additionally, when tissue expanders are typically removed from their subpectoral position in delayed-immediate reconstruction, the pectoralis major is repositioned on the chest wall so that the DIEP flap is placed appropriately on top of the muscle, which leads to more optimal aesthetic outcomes [38]. We utilize all or some of these proposed sutures depending on the needs of the reconstruction to provide stability of the flap and further improve the shape of the breast mound following the purse-string DIEPplasty. Of note, when placing these sutures, surgeons should avoid tying the sutures down too tight or trapping any fat within the knot to decrease the risk of fat necrosis.

**Figure 9:**
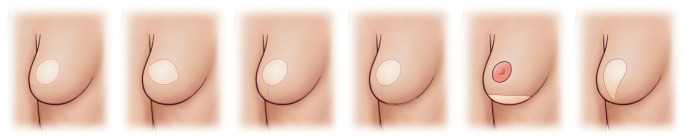
Illustration for DIEP flap skin paddle pattern. Various skin paddle designs can be utilized to optimize inset and integration with the mastectomy skin envelope, depending on reconstructive goals and skin quality. Thoughtful selection of skin paddle pattern facilitates contour refinement, symmetry, and aesthetic blending of the reconstructed breast. Paddle design is matched to skin quality and envelope defect, which governs scar visibility and how flap skin blends with native skin.

Another important intraoperative consideration pertains to the eventual DIEP flap skin paddle design and inset (Figure 9). There have been various techniques described, such as a small elliptical or NAC-shaped skin paddle positioned in the center of the breast within the mastectomy scar or a vertical ellipse skin paddle extending from the mastectomy incision scar to the IMF [39]. The choice of skin paddle design is also directly influenced by the radiation changes and the subsequent required excision patterns based on the parts of the skin envelope that are damaged. Specifically, in cases where radiation has caused significant fibrotic changes to the skin envelope, this is typically excised, and the inferior part of the DIEP flap skin paddle becomes the upper part of the recreated inframammary fold [38]. Some authors have also recommended incising the dermis around the skin paddle prior to closure to provide a smoother transition between the mastectomy and flap skin and to avoid any indentations and possible scar bands [13]. Regardless of the techniques used, it is critical for every plastic surgeon to be meticulous and systematic when evaluating the aesthetics of the DIEP flaps during flap inset. This includes sitting the patient up during this part of the surgery to ensure ideal symmetry. In every case, one drain is typically placed in each reconstructed breast to facilitate drainage.

#### 3.2.5. Abdominal Wall Closure Considerations

**Figure 10:**
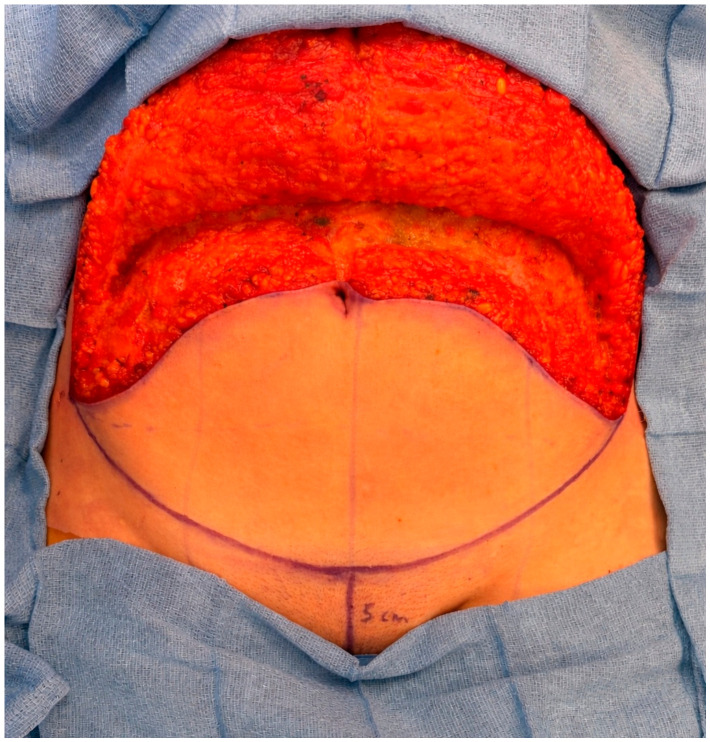
Intraoperative photograph of abdominal donor site incision. The incision pattern maintains low placement of the upper incision to optimize scar concealment and cosmesis.

When addressing the abdomen in DIEP flap reconstruction, the common theme is to approach it similarly to an abdominoplasty to optimize aesthetic outcomes at the donor site. Some of the most common aesthetic causes of patient dissatisfaction include the scar location, uneven abdominal wall thickness superior and inferior to the scar, residual abdominal overhang, and bulging or dog ears on the lateral edges of the incision [39,148]. Many of these can be mitigated during the initial surgery with meticulous attention to detail and certain considerations that are individualized to each patient’s anatomical needs. When designing the upper and lower incisions on the abdomen, we follow the same principles whenever possible to produce reliable and reproducible aesthetic outcomes. We always ensure that the lower transverse scar is as low as possible so that it is hidden well in the underwear line (Figure 10). We also try to keep the upper incision low and sometimes even inferior to the umbilicus in order to facilitate a lower overall position of the final scar. If we keep the upper incision low and inferior to the umbilicus, the upper flap dissection is done meticulously with significant superior beveling to preserve as much adipose tissue as possible and protect any prominent perforators in that area (Figure 10). Applying these principles is more feasible in patients with significant abdominal tissue and adiposity but can often be challenging in patients that lack sufficient abdominal laxity. There have been several flap designing techniques described in the literature for these situations, such as mini-DIEP flap and the low DIEP flap for reconstruction of small to moderately sized breasts [39,149,150,151,152]. Moreover, cases in which it is deemed necessary to harvest both medial and lateral perforators to support flap perfusion are often associated with more extensive dissection of abdominal muscle. Clark et al. recently described the APEX, or abdominal perforator exchange, technique involving dissection of these intramuscular perforators at their origins and re-anastomosing vessels in the supramuscular plane to minimize morbidity to musculature and nerves when doing so. APEX flaps have shown favorable outcomes and improved sparing of the rectus muscle and have been recommended when it is expected that more extensive sacrifice of the rectus muscle or splitting of motor nerve branches is needed for pedicle isolation in a specific patient [63].

The low DIEP flap is one such approach that aims to place the abdominal scar as low as possible on the abdomen to best facilitate concealment and improve cosmesis. Eom et al. published a technique paper describing patient selection, stating that the procedure is indicated in cases where a dominant, reliable perforator is found more than 4 cm below the umbilicus. Moreover, the low DIEP flap is described as being smaller than the conventional DIEP and is therefore best suited for patients with small-to-moderately sized breasts [150]. In terms of operative technique, the approach has been applied successfully. This same group showed that the abdominal scar is much more favorably positioned, close to the pubic rim, and could be well concealed with underwear. While aesthetic outcomes are favorable, the low DIEP approach is more susceptible to flap failure resulting from venous congestion due to selection of inadequate perforators and thus requires heightened care in interpreting pre-operative angiography [151]. Indeed, a 2019 study reported on 103 patients who underwent low DIEP flap breast reconstruction and showed aesthetically superior outcomes with lower abdominal scars and natural navel positions. The scar was, on average, 0.6 cm above the pubic hairline and 0.4 cm below the anterior superior iliac spine, with the umbilicus positioned 7.0 cm above the pubic hairline [153].

**Figure 11:**
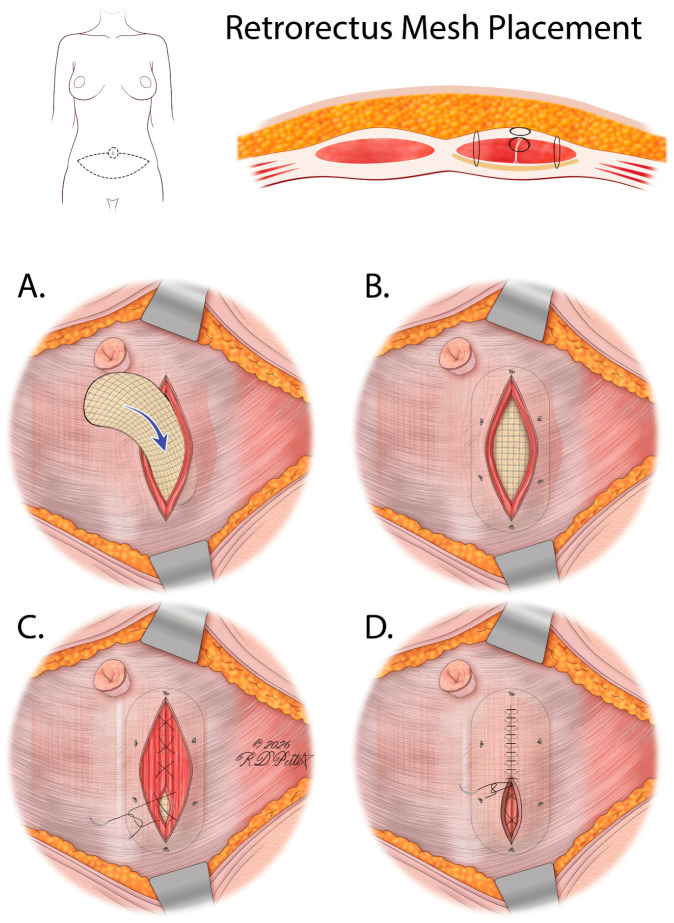
Illustration of Ovitex in retro-rectus plane. Placement of reinforced biosynthetic mesh within the retro-rectus plane provides structural support to the abdominal wall following DIEP flap harvest. This technique reduces the risk of bulge and hernia while preserving abdominal contour and optimizing donor site aesthetics. (**A**) Placement of oval shaped Ovitex in retro-rectus plane; (**B**) Ovitex is secured to the anterior rectus sheath with horizontal mattress 2-0 Vicryl sutures, which are passed through the rectus abdominis muscle, ensuring appropriate tension; (**C**) The anterior rectus sheath is closed in two layers, first with long absorbing buried figure-of-eight sutures; (**D**) followed by a running barbed long absorbing suture.

An important consideration during abdominal wall closure includes some of the undesirable complications that are often seen after DIEP flap reconstruction, such as abdominal wall bulges and hernias, with abdominal wall bulge rates reported anywhere from 0% to 33% in the literature and abdominal hernia rates ranging from 0% to 7.1% [154,155,156,157,158]. The use of synthetic meshes like polypropylene mesh to reinforce the abdominal wall fascia after DIEP flap harvest gained traction over the years due to its high success in reducing bulge and hernia rates [154,159,160,161]. Despite their success in bulge and hernia prevention, these synthetic meshes were associated with seromas and infections that often required surgical removal [162,163,164]. Based on the available literature and our senior authors’ experience, our institution has adopted the use of the Ovitex Core reinforced tissue matrix to reinforce the abdominal wall fascial defect following DIEP harvest since it has the added benefits of promoting wound healing and avoiding foreign body response while also being effective in decreasing the risk of bulges and hernias [159,164]. This Ovitex mesh is shaped as an oval and subsequently placed as a sublay in the recto-rectus space and secured to the anterior rectus sheath with horizontal mattress 2-0 Vicryl sutures, which are passed through the rectus abdominis muscle, ensuring appropriate tension (Figure 11). We subsequently close the anterior rectus sheath in two layers with long absorbing buried figure-of-eight sutures followed by a running barbed long absorbing suture. A study that evaluated the efficacy and safety of the Ovitex mesh compared 85 patients who had abdominal wall reinforcement with Ovitex to 114 patients that did not have any mesh reinforcement and found that the use of Ovitex was associated with development of fewer abdominal bulges (0% vs. 5.3%, *p* = 0.04) compared to the non-mesh cohort and did not increase the rate of adverse events such as hematomas and seromas [159]. Other absorbable meshes, such as poly-4-hydroxybutyrate (P4HB), have also been used with promising outcomes. In a study by Wormer et al., 160 patients undergoing DIEP reconstruction with biosynthetic mesh onlay (Phasix [monofilament poly-4-hydroxybutyrate], Bard Inc., Warwick, RI) for anterior rectus fascia reinforcement were compared to 159 patients who did not have a mesh placed, and their findings showed that the placement of the mesh reduced the bulge rate in the mesh cohort compared to the non-mesh cohort (0 vs. 5.5%; *p* = 0.008) [165]. Another recent retrospective study by Silverman et al. compared 59 patients who underwent DIEP flap reconstruction with retrorectus P4HB mesh placement to 59 patients who did not have any abdominal wall reinforcement and showed that the bulge rate was lower in the mesh group compared to the non-mesh group (5.1 vs. 18.6%; *p* = 0.04), while there were no statistically significant differences in hernia, seroma, or infection rates between the two groups [166]. Other surgeons have advocated for microfascial incisions limited to 2 cm on average when harvesting DIEP flaps with precise fascial repair and no mesh placement to minimize donor site morbidity [167]. While this is a good approach for small-to-moderate-sized flaps, it might be a lot more challenging to achieve for a large flap harvest requiring the harvest of more than one perforator. More broadly, the use of mesh has been well established as a useful adjunct in both implant-based reconstruction, where it is used more in the breast to support the tissue expander or implant, and in DIEP flap reconstruction, where it is used both to support abdominal wall reinforcement and the breast. One important limitation is cost, which varies substantially between products and institutions. Publicly available pricing data have reported AlloDerm at approximately $30.80/cm^2^, whereas GalaFLEX, a P4HB-based synthetic absorbable mesh, has been reported at approximately $16–18/cm^2^. Ovitex has additionally been reported at approximately $19/cm^2^ [168]. Surgeon preference for one mesh over another will ultimately depend on the continued emergence of high-quality comparative data and on individual experience in DIEP flap reconstruction.

Moreover, robotic surgery has also been added to the armamentarium of plastic surgeons with promising results about its safety and reliability in minimizing donor site morbidity by limiting the length of the fascial incision [169,170]. A recent systematic review conducted by Morkuzu et al. corroborates earlier work finding reduced postoperative pain and donor site morbidity relating to smaller fascial incisions in the robotic DIEP cohort. Approaches include the totally extraperitoneal (TEP) approach or the transabdominal pre-peritoneal (TAPP) approach for flap harvest, with TEP being less invasive by preserving the posterior rectus sheath but having a steeper learning curve [171]. Robotic DIEP harvest has been shown to reduce narcotic intake and pain during the early postoperative period [172]. With similar complication rates, a separate systematic review shows robotic DIEP surgery is associated with a significantly prolonged operative time but a significantly reduced length of stay [173]. Learning curve analyses have shown that incorporating robotic DIEP surgery into a practice is expected to require approximately 10 flap harvests for the plastic surgeon to attain proficiency [174]. Overall, more research is required to better characterize the impact of robotic surgery on aesthetic outcomes in DIEP flap reconstruction and on the implications of cost on broader adoption.

The positioning of the umbilicus is another important aesthetic consideration. There is a lot of debate in the literature about what constitutes the most aesthetically pleasing umbilicus in DIEP flap reconstruction. Many studies have shown that a vertical or oval-shaped umbilicus with no protrusion and superior hooding leads to more optimal aesthetic outcomes [175,176]. Regardless of the technique used, surgeons typically tailor-tack the abdominal incision with staples prior to definitive closure to ensure appropriate placement and symmetry of the umbilicus either along a pre-determined position in the midline or at the level of the iliac crest [39,177]. Some authors have recommended using the golden ratio of the abdominal aesthetic unit, defined as the ratio of the xiphoid-umbilicus distance to the umbilicus-abdominal crease distance in order to determine the ideal position of the umbilicus [39,175]. Umbilical incisions can be designed in many different ways, including vertical ellipse, round, inverted U, or U-shaped [39]. We typically prefer an inverted U-shaped incision with excision of a wedge from the inferior aspect of the umbilicus, similar to other authors’ preference [13,178]. Of note, defatting the umbilical stalk is common in most techniques, while others have proposed tacking the umbilical stalk to abdominal fascia to provide a more aesthetically pleasing contour [13,39]. We do utilize both these approaches depending on the patient’s anatomy, appearance of the native umbilicus, and thickness of the remaining abdominal wall tissue.

**Figure 12:**
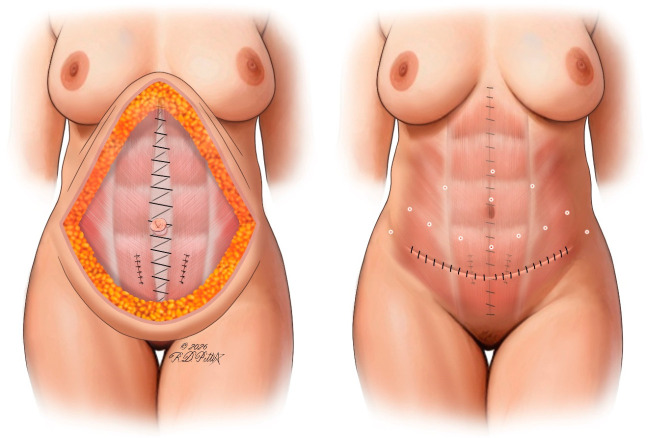
Illustration of rectus diastasis repair. Midline plication of the rectus abdominis muscles restores abdominal wall integrity following DIEP flap harvest, improving core function and abdominal contour. Restoration of midline tension eliminates central abdominal bulging, addressing both donor-site function and aesthetics. Also depicted are progressive tension sutures (white dots), which reduce dead space, distribute closure tension, and facilitate earlier drain removal while helping optimize scar quality and abdominal contour.

Prior to abdominal closure, there are some important steps that can optimize aesthetic outcomes. While plication has traditionally been performed at a later stage in response to significant diastasis or laxity, increasing evidence is showing the benefits of taking a proactive approach [179]. Other research has investigated pedicle dissection techniques, such as the medial paramuscular approach, which has been shown to reduce the incidence of abdominal bulging compared with lateral perforator harvesting [180,181]. Others have demonstrated favorable results by combining all the aforementioned approaches, performing a low-incision drainless DIEP flap in combination with corsetplasty and diastasis repair [182]. Rectus plication can improve abdominal contour and is often an important aspect of donor site optimization in DIEP flap patients. However, because rectus diastasis can vary widely in presentation and severity, no single repair strategy is universally applicable. Keramidas et al. proposed a four-type classification system and treatment algorithm for rectus diastasis correction based on the severity of diastasis and abdominal wall deformity in a cohort of patients undergoing abdominoplasty. The authors reported favorable patient-reported aesthetic outcomes, minimal pain, low complication rates, and no clinical recurrence during follow-up using individualized plication strategies [183]. While this presents an important framework for considering rectus diastasis and repair planning, the considerations in DIEP flap donor site optimization differ considerably. In DIEP flap patients, the lower abdominal fascia is already partially plicated and altered following DIEP flap harvest. Thus, only supraumbilical plication is often required in these patients, with some infraumbilical extension at times. Moreover, special circumstances such as unilateral flap harvest may require asymmetric plication that is not well represented in other clinical contexts. Further work is warranted to better characterize rectus diastasis and optimal management strategies among DIEP flap patients. In our senior authors’ experience, rectus plication in two layers can lead to a more aesthetic abdominal contour and is especially crucial in rectus diastasis cases. In such cases, we typically use 0 polydioxanone buried interrupted figure-of-eight sutures to plicate the supraumbilical and sometimes the infraumbilical rectus diastasis, followed by a running 2-0 polydioxanone Stratafix suture to reinforce the plication with consistent tension (Figure 12). In addition, we also use progressive tension sutures with interrupted 2-0 Vicryl sutures, which have been shown to decrease the tension on the abdominal closure, prevent scar widening, and avoid wound dehiscence [39,184]. We have also noticed that with the use of progressive tension sutures, the abdominal skin flap incorporates faster, allowing earlier removal of drains without increasing the risk of seromas. This has also allowed us to use one drain at times. Other authors have advocated for a running-barbed quilting technique under tension between the abdominal skin flap and the abdominal wall to maximize obliteration of dead space and decrease overall drainage [39,185]. Regardless of the technique used, the overarching goal is to achieve a tension-free closure with good wound edge eversion to optimize healing and aesthetic outcomes. The incisions should typically be closed from lateral to medial, and whenever necessary, extend the incision laterally to avoid dog ears [39]. One or two drains are placed in the donor site. Some other authors, such as Nagarkar et al., advocate that drains are not necessary when progressive tension sutures are utilized since the complication rates are not statistically different [186].

### 3.3. Approach to DIEP Revision

#### 3.3.1. General Principles in DIEP Flap Revision

As previously discussed, most DIEP flap patients undergo revision surgeries [187]. Despite meticulous pre-operative planning and intraoperative execution, revision surgeries are commonly required to achieve the most aesthetically pleasing result given the high expectations of this patient population. Research has shown that among TRAM and DIEP patients, the most common reasons for additional surgery were aesthetic in nature. The rates of elective revision surgery following breast reconstruction remain high, with as many as 40.2% of patients with no complications electing to undergo revision surgeries, requiring a mean total of 2.2 surgeries to achieve a stable and satisfactory reconstruction [188]. DIEP flap reconstruction patients are among the most likely to undergo revisions compared to implant-based reconstruction or other flap types including transverse rectus abdominis myocutaneous (TRAM) and latissimus dorsi (LD) flaps [188]. Thus, there is still room for progress and fine-tuning of the autologous breast reconstruction process to improve cosmetic outcomes.

Various studies have described corrective procedures after mastectomy and DIEP flap reconstruction, including reshaping and correction of ptosis, nipple reconstruction, and implant insertion, among others [189]. One study found the most common reason for revision was flap revision for shaping (42.0%), followed by nipple reconstruction (26.9%) and abdominal deformity correction (25.2%) [21]. Smith et al. found that among a cohort undergoing abdominal-based free flap reconstruction, 903 of 1251 patients (72.2%) underwent at least one revision procedure, with an average number of revisions specifically performed for aesthetic concerns being 1.1 ± 0.9 overall among this cohort [190]. These revision procedures are not a reflection of technical failure; rather, they represent an expected component necessary for optimizing long-term cosmesis once flaps have fully incorporated and postoperative swelling has resolved. Revisions provide an opportunity to refine and enhance breast shape, excess skin or residual ptosis, NAC appearance and position, abdominal contour, and other recipient and/or donor site aesthetic outcomes. This should be discussed with patients pre-operatively to set appropriate expectations.

#### 3.3.2. NAC, Excess Skin, and Persistent Ptosis Revision

**Figure 13:**
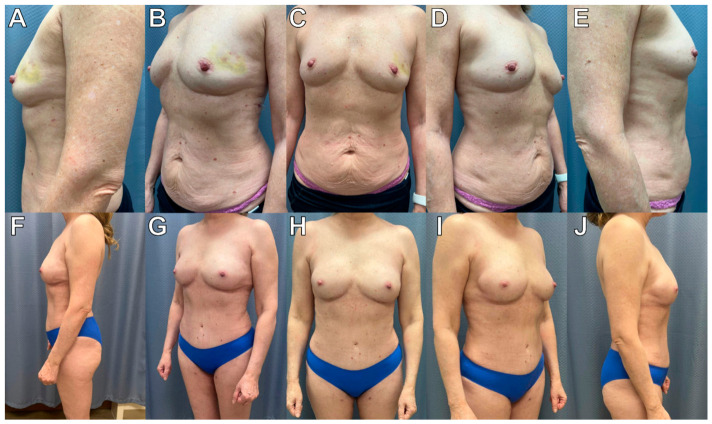
Pre- and postoperative photographs of post-DIEP revision mastopexy in NSM. A 60-year-old patient with left breast invasive lobular carcinoma underwent bilateral NSM via inframammary incisions followed by staged reconstruction and eventual DIEP flaps. Purse-string DIEPplasty technique enabled enhanced projection and refined breast boundary definition, contributing to an improved aesthetic outcome. (**A**–**E**) Pre-operative photographs. (**F**–**J**) Postoperative photographs obtained 9 months after mastopexy and fat grafting.

Residual skin laxity, malposition of the NAC, asymmetry, and general breast ptosis are common indications for revision mastopexy following DIEP flap reconstruction. The choice of mastopexy approach depends on several factors including surgeon preference, perfusion characteristics of the reconstructed breast and NAC, and the dermal-DIEP flow pattern. Traditionally, the principal concern with post-DIEP mastopexy was that perfusion of the NAC primarily comes from the DIEP flap and is particularly susceptible to disruption and necrosis, particularly in the case of reoperation [73]. For patients experiencing persistent ptosis, laxity, or NAC asymmetry following DIEP flap reconstruction, the surgeon must carefully plan revision mastopexy to avoid any compromise of NAC perfusion. Notably, DellaCroce et al. described a technique in which NSM and DIEP flap reconstruction was performed in patients with grade II or III ptosis, followed by secondary mastopexy after an average of 185 days. The authors described the use of various incision patterns at the time of mastopexy, ranging from Wise to circumvertical, depending on the degree of ptosis and breast shape. A full-thickness, complete periareolar incision with de-epithelialization is performed, followed by entry into the subcutaneous plane and controlled undermining of the overlying breast skin from the underlying perforator flap. This allows for redraping of the skin envelope, contour modification, and mobilization of the NAC to the desired position. Notably, the NAC is repositioned without reliance on a fixed pedicle, instead supported by perfusion from the underlying flap and surrounding tissues. This technique was successfully applied to 70 patients who underwent 116 NSMs, yielding high patient satisfaction and low complication rates, including in patients with grade III ptosis and macromastia. This study demonstrates principles which are applicable to revision mastopexy. Notably, with proper technique, even unplanned revision mastopexy for asymmetry, residual ptosis, or laxity can be safely performed with significant mobilization of the NAC using vascular contributions from the underlying flap [75]. This is the authors’ preferred approach given its versatility, reliability, and maintenance of satisfactory long-term results (Figure 13).

A more recent study described a similar Wise pattern mastopexy technique during revision performed in three patients who underwent NSM with DIEP flap reconstruction and required reshaping of the breast and correction of persistent ptosis. Following Wise pattern marking of the breasts, the authors describe de-epithelialization of the Wise pattern and subsequent superior, near-complete dissection of the NAC, maintaining perfusion primarily through the DIEP and inferior mastectomy flaps, permitting safe repositioning of the NAC and reshaping of the reconstructed breasts while maintaining the integrity of DIEP pedicles and NAC perfusion as confirmed via intraoperative SPY fluorescence using indocyanine green angiography. This approach permitted safe optimization of aesthetic outcomes in all three patients [73]. One report details the case of a patient who underwent NSM and DIEP flap reconstruction and continued to experience persistent asymmetry with the NAC positioned 2.5 cm lower on the right side. Symmetrizing revision involved the use of a ‘reinforced pedicle technique’ in which the NAC was elevated on a reinforced superior pedicle of the mastectomy flap in combination with the underlying DIEP flap, thus maintaining dual blood supply. This approach permitted safe elevation of the NAC by 2.5 cm without encountering further complications [191]. Others have still described other techniques, including crescentic mastopexy with or without temporal delay to allow for more robust neovascularization of the NAC following DIEP flap reconstruction if significant mobilization of the NAC is required as part of revision [72,192].

#### 3.3.3. Fat Grafting in DIEP Flap Reconstruction

Autologous fat transfer has broad application in breast reconstruction and is a valuable adjunct in autologous breast reconstruction to maximize aesthetic outcomes. It is favored for its ability to correct contour irregularities, size asymmetries, and superomedial breast concavities resulting from exposure of the recipient internal mammary vessels [193,194]. Fat grafting has also been shown to improve outcomes in patients with lower body mass index and thin body habitus with larger breast sizes [195]. Other indications explored in the literature include correction of insufficient volume in otherwise symmetrical breasts and correction of irregularities due to contracture [196]. For example, it can be utilized to increase the volume of a flap during a revision and avoid the use of stacked flaps from different donor sites or the use of implants during the initial operation. When dealing with contractures and scarring, release with subcision prior to fat grafting may be required, and this is something that we routinely perform in our practice. Moreover, autologous fat transfer has been proposed to address fat necrosis through several mechanisms including breaking down areas of fibrotic tissue, improving local tissue conditions through remodeling, and improving cosmesis [194]. Most post-DIEP fat grafting described in the literature is performed after reconstruction, ranging from 3–4 months postoperatively to 98 months after primary reconstruction [196,197,198]. However, the use of simultaneous DIEP flap reconstruction and fat grafting has been described. Specifically, Hartrampf zone IV of the DIEP flap, which is often discarded due to its distance from the perforator vessels in zone I and risks of partial flap loss and necrosis, can be used as a donor site for autologous fat transfer. Favorable cosmetic results have been achieved using this technique to correct contour irregularities, particularly around the décolleté line of the upper pole, both in DIEP and TRAM flap reconstruction [199,200]. Other than the DIEP flap itself, other donor sites used for autologous fat transfer include the abdomen, such as from Holm zone IV, which can facilitate simultaneous DIEP dissection and fat harvesting, the medial and lateral thighs, and the flanks with volumes up to 240 cc [19,197]. Depending on the size of the breasts and the amount of autologous fat the tissues can accommodate, even higher volumes of fat transfer are sometimes used in our operating rooms, but it is always done as a secondary stage.

Overall outcomes described in the literature regarding the use of autologous fat transfer in the context of DIEP flap reconstruction are favorable. This adjunctive measure has been shown to be effective in irradiated breasts and results in retention of as much as 70.9% of the lipofilling projection achieved immediately after grafting, with a reported mean integration rate of 45.98% [197,201]. Subjective aesthetic outcome measures have also been favorable; fat grafting has been shown to significantly improve breast volume and shape subjective measures (*p* < 0.001) as well as patient-reported satisfaction with projection (*p* = 0.00782), medial contour (*p* = 0.0268), and overall appearance (*p* = 0.00769) compared with no fat grafting after reconstruction [19]. Indeed, other research comparing flap reconstruction cohorts with a diverse array of flaps including free DIEP and pedicled latissimus dorsi flaps, either without or with fat grafting (mean volume = 88 mL), utilized the BREAST-Q to compare groups and found a significantly improved “Satisfaction with outcome” score in the fat grafting group (86 ± 8.0 vs. 79 ± 9.5, *p* = 0.020) [196]. One systematic review including 43 studies and 6260 patients found an overall volume retention of 76.8%, an overall patient satisfaction rate of 93.4%, and a surgeon satisfaction rate of 90.1% [195]. Emerging adjuncts have demonstrated promise in enhancing the efficacy of fat grafting by improving graft survival. Stromal vascular fraction (SVF), for example, is processed from a patient’s own adipose cells and has been shown to significantly improve graft retention without increasing complication rates [202]. This improvement is sustained through one year postoperatively and is not associated with an increased risk of locoregional recurrence in the context of autologous breast reconstruction [203]. Similarly, other modalities of enhanced fat grafting, such as the use of cell-assisted lipotransfer (CAL) and platelet-rich plasma (PRP), have shown a significant benefit in graft survival without an increase in complication rates [204]. However, while these adjuncts may have a significant potential benefit to the aesthetic outcome in breast reconstruction, their safety profile has not yet been well characterized, which limits broader adoption [205].

While the oncologic safety of fat grafting remains an area of investigation, current evidence has generally shown no significant increases in recurrence risk [194]. One systematic review found an average locoregional and distant oncological recurrence of 2.5% and 2.0%, respectively, after fat grafting with an overall acceptable complication rate not suggestive of increased risk of recurrence associated with this procedure. However, the authors did find that fat grafting resulted in more biopsies performed based on radiological findings (3.7% vs. 1.6%) and more cases of fat necrosis (9.0% vs. 4.7%) [195]. Conversely, one matched retrospective cohort study assessing disease-free survival between 100 patients who underwent DIEP flap reconstruction with lipofilling and 100 matched patients who underwent only DIEP flap reconstruction showed that lipofilling increased recurrence risk in patients with positive nodal status (*p* = 0.035) and a high-grade neoplasia (*p* = 0.049) [198].

#### 3.3.4. Revision of the Abdomen

Both breast and abdominal scars can significantly influence patient satisfaction with aesthetic outcomes and overall quality of life following DIEP flap breast reconstruction [149,206]. In the context of abdominal scars specifically, the DIEP flap has the most extensive donor site scar and was the least favored among a random survey of five hundred men and women [207]. In a cohort of 248 patients who underwent DIEP flap reconstruction a mean of 2.6 ± 1.9 years prior, 38% reported ongoing abdominal symptoms, including issues related to scarring. Moreover, compared to women without abdominal scars, women with abdominal scars had BREAST-Q scores that were, on average, 11 and 13.2 points lower (*p* ≤ 0.001) in the sexual and physical well-being domains, respectively. Among all included patients, 40.3% reported being very or somewhat dissatisfied with their abdominal scars, and 13.7% reported the same regarding the position of their navels [208].

The first and most crucial step in planning to mitigate the influence of postoperative scars on patient satisfaction is to ensure patients are adequately counseled on the possible range of scar outcomes following DIEP flap reconstruction. Everaars et al. showed that much of the postoperative dissatisfaction that patients felt following DIEP flap breast reconstruction came from inadequate pre-operative patient counseling. “They never tell you anything about the scars”, one patient reported, while another said, “Yes, they did, but not how to massage them…but how to do this? I really have no idea. I was never told how to do that” [206].

Outside of prophylaxis described in previous sections, novel techniques have also been described to manage scars that have already occurred. Traditional approaches include invasive revision surgery for scar revision, but there has been an increase in the use of noninvasive alternative approaches that leverage the body’s own regenerative mechanisms. One randomized controlled split-trial was conducted on 30 patients who had abdominal scars following DIEP flap reconstruction. One side was treated with microneedling, and the other served as a control. Using the Patient and Observer Scar Assessment Scale (POSAS) instrument, the treatment side showed significantly lower scores at 3- and 9-month follow-up (17, IQR = 18.3 treated vs. 21.4, IQR = 17.5 untreated, z = −2.1482, *p* = 0.01). POSAS items, including overall opinion and all scar characteristics including itch, stiffness, thickness, and irregularity, were all significantly improved on the treated side [209].

## 4. Limitations

Several limitations should be considered when interpreting this review. First, this manuscript represents a narrative review integrating a targeted literature search coupled with consensus-based recommendations made by our senior authors. This study does not represent a formal systematic review with meta-analysis. This approach allows a nuanced discussion of operative decision-making and experience with a variety of approaches in DIEP flap reconstruction that may not be captured by a formal literature search covering topics without a strong body of evidence. However, this approach may also introduce inherent selection bias in the topics emphasized, the studies discussed, and the recommendations made. Additionally, much of the available literature covering aesthetic optimization in DIEP flap reconstruction remains retrospective, heterogeneous, and technique-dependent, with relatively limited high-level prospective data available for many reconstructive approaches discussed throughout this review. As such, certain recommendations and technical preferences described throughout this paper represent still evolving concepts supported by variable levels of scientific evidence and by our expert consensus at a high-volume breast reconstruction center. Further evidence driven by rigorous and controlled investigation is required to more definitively demonstrate the superiority of one approach over another. Finally, many emerging topics, including robot-assisted harvest, AI-based, VR-based, and AR-based planning and navigation, neurotization, and regenerative adjuncts and biomaterials including mesh, lack clear evidence relating to long-term aesthetic outcomes and cost-effectiveness, and similarly require further research.

## 5. Conclusions

The DIEP flap remains the gold standard for autologous tissue breast reconstruction, with continued advancements gradually shifting the focus from flap survival and patient safety to optimization of aesthetic and patient-centered outcomes. Achieving optimal cosmesis requires a comprehensive approach spanning pre-operative planning, mastectomy technique and nipple–areolar complex management, reconstructive timing and staging, meticulous intraoperative execution, and selective incorporation of revision surgeries for aesthetic refinement. As techniques, technologies, and the body of evidence continue to evolve, a personalized and aesthetically driven approach remains essential to advancing the quality and consistency of outcomes in autologous breast reconstruction.

## Data Availability

No new data were created or analyzed in this study. Data sharing is not applicable to this article.
